# Supplementary material for: Integrative Metabolomics and Proteomics Allow the Global Intracellular Characterization of Bacillus subtilis Cells and Spores
Source: J Proteome Res. 2024 Jan 8;23(2):596–608. doi: 10.1021/acs.jproteome.3c00386 (PMC10845140; doi:10.1021/acs.jproteome.3c00386)
Supplement: Supplementary file 1 — pr3c00386_si_001.pdf [file pr3c00386_si_001.pdf]

Supporting information

Integrative metabolomics and proteomics allow the global intracellular characterization of *Bacillus subtilis* cells and spores.

Yixuan Huang [1,2], Bhagyashree N. Swarge [1,2†], Winfried Roseboom[1], Jurre Bleeker[1], Stanley Brul[2], Peter Setlow[3], Gertjan Kramer [1,*]

[1]*Laboratory for Mass Spectrometry of Biomolecules, Swammerdam Institute for Life Sciences, University of Amsterdam, Science Park 904, 1098 XH Amsterdam, The Netherlands*

[2]*Molecular Biology & Microbial Food Safety, Swammerdam Institute for Life Sciences, University of Amsterdam, Science Park 904, 1098 XH Amsterdam, The Netherlands;*

[3]*Department of Molecular Biology and Biophysics, UConn Health, Farmington, CT 06030-3305 USA;*

[†]Current address: *Department of Biotechnology, Delft University of Technology, Delft, the Netherlands*

[*] *Correspondence: G.Kramer@uva.nl (G.K.)*

Table of Content

Supplemental figures

Figure S1: Evaluation of metabolite data quality.

Figure S2: Relative representation of molecules extracted by 60% EtOH and 1-propanol.

Figure S3: Evaluation of proteome data quality.

Supplemental tables

The supplemental tables listed below can be found in separated Excel files.

Table S1: Comparison of annotated metabolites extracted by 60% EtOH and 1-propanol.

Table S2: Proteins identified and quantified in *B. subtilis* cells.

Table S3: Proteins identified and quantified in *B. subtilis* spores.

Table S4: Annotated metabolic features in *B. subtilis* cells and spores.

Table S5: All metabolic features in *B. subtilis* cells and spores.

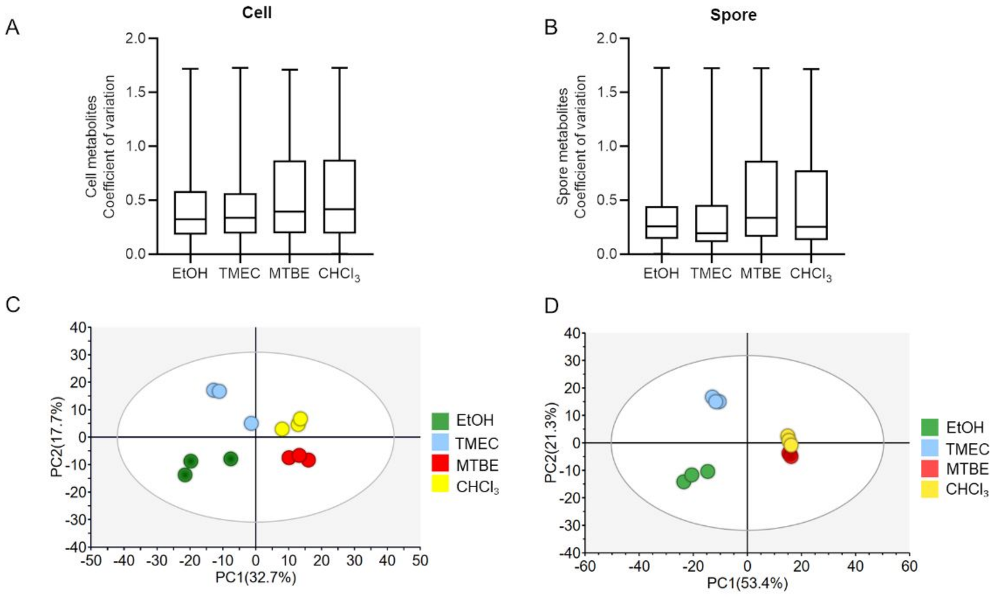

Figure S1. Evaluation of metabolite data quality using different extraction methods for *B. subtilis* cells and spores. A and B, box plots of coefficient of variation (cv) across all the features identified in samples extracted by different methods. C and D, PCA of B. subtilis cells and spore's identified metabolites respectively with different methods.

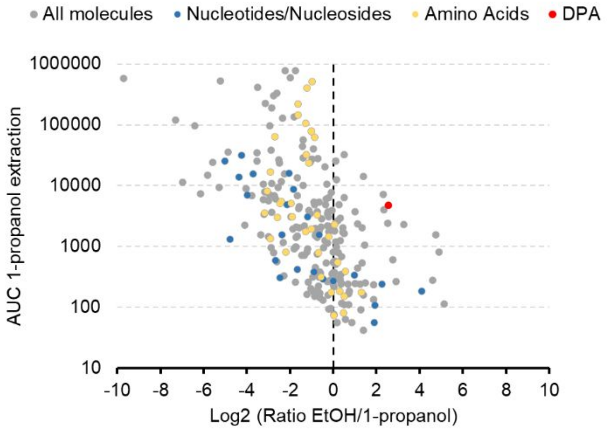

Figure S2. Relative representation of molecules extracted by 60% EtOH with mechanical disruption to boiling 1-propanol extraction in *B. subtilis* spores. X-axis shows ratio between AUC of the two extractions showing molecules with predominant signals in one of the two extractions, Y-axis shows the AUC in the 1-propanol extraction showing the signal intensity of the various molecules in that extraction.

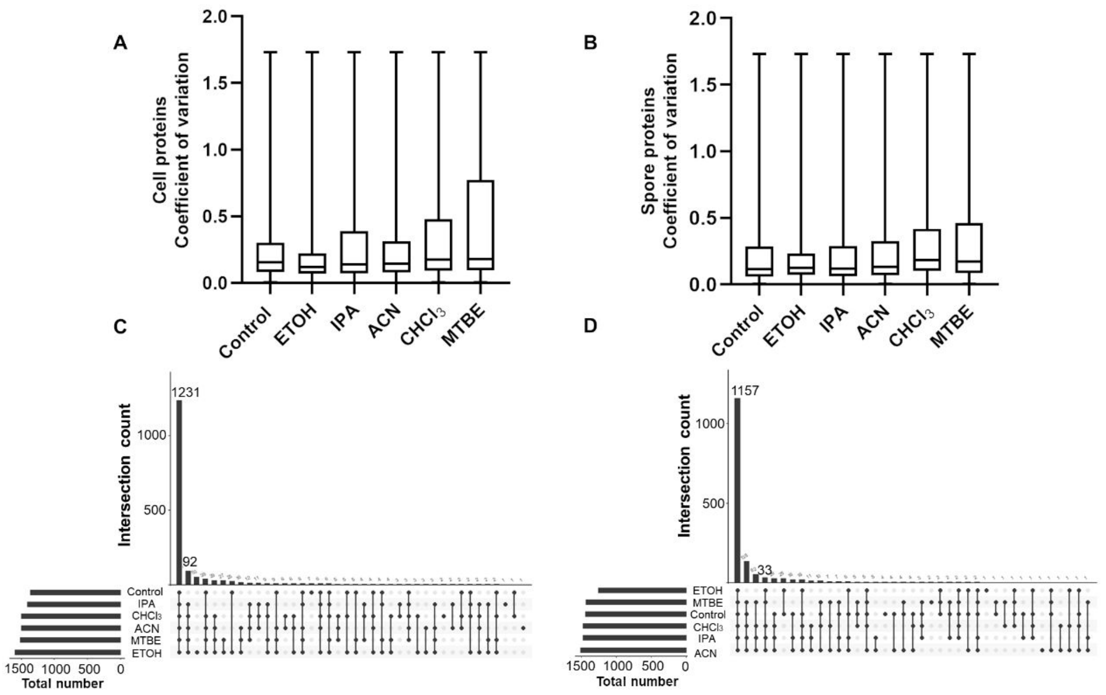

Figure S3. Evaluation of proteome data quality using different extraction methods in *B. subtilis* cells and spores. Control group was lysis by 1% SDS without previous extraction. The A and B, box plots of coefficient of variation (cv) across proteins identified in samples extracted by different methods from B. subtilis cells and spores. C and D upset diagram showing the overlapping (shared) and unique metabolites identified in cells and spores by different extraction methods and normal extraction for proteomics.
